# 

**Published:** 1890-05

**Authors:** 


### BOOKS AND PAMPHLETS RECEIVED.

Bulletin of the American Museum of Natural History (Central Park, New York). Vol. II, Nos. 3–4.

Bulletin Menseul Société Linneenne du Nord de la France. Amiens.

Bulletin de la Société Zoologique de France. Paris.

Bulletin de L'Academie Royale de Médecine. Brussels.

Memorie della R. Accademia Delle Scienze dell' Istituto di Bologna. Serie IV. Tomo IX.

Atti della R. Accademia Delle Scienze di Torino. Vol. XXIV. 1888–89.

Journal and Proceedings Royal Society New South Wales. Vol. XXIII., Pt. I. Sydney.

Annalen des K. K. Naturhistorischen Hofmuseums. Bd. IV, No. 4. Bd. V. No. 1. Wien.

Veileder fur Besogende i den Zoologiske Have ved Kjobenhavn. 1889.

Smithsonian Reports. 1886–87. 4 vols. Washington, 1889.
Bulletin U. S. Fish Commission. Vol. VII. 1887. Washington, 1889.
Transactions Kansas Academy of Science. Vols. VIII–IX. 1881–84. Topeka.
Journal Cincinnati Society of Natural History. Vol. XII, No. 4.
"The Ornithology of Illinois." Pt. I. Descriptive Catalogue. By Robert Ridgway. Part II. Economic Ornithology. By S. A. Forbes. Vol. I. Published by authority of State Legislature. Royal 8vo, pp. 520. 32 pp. plates. Springfield, 1889.
"A Descriptive Catalogue of the Phalangiinæ of Illinois and a Partial Bibliography of the Phalangiinæ of North America." By Clarence M. Weed. From the *Bulletin Illinois State Laboratory of Natural History.* Vol. III. Champaigne.
"The Relation of the Thalmus to the Paracoele (Lateral Ventricle)." By Burt G. Wilder. Reprinted from the *Jour. Nervous and Mental Disease.* July, 1890.
"On the Position of Chamœa in the System." By R. W. Shufeldt. Reprinted from the *Jour. of Morphology.* Vol. III, No. 3.
Il Naturalista Siciliano.
Ottawa Naturalist.

